# Effect of long-term cold storage on trehalose metabolism of pre-wintering Harmonia axyridis adults and changes in morphological diversity before and after wintering

**DOI:** 10.1371/journal.pone.0230435

**Published:** 2020-03-19

**Authors:** Boping Zeng, Shasha Wang, Yan Li, Zhongjiu Xiao, Min Zhou, Shigui Wang, Daowei Zhang

**Affiliations:** 1 School of Biological and Agricultural Science and Technology, Zunyi Normal University, Zunyi, Guizhou, China; 2 College of Life and Environmental Sciences, Hangzhou Normal University, Hangzhou, Zhejiang, China; Chinese Academy of Agricultural Sciences Institute of Plant Protection, CHINA

## Abstract

*Harmonia axyridis* is a major bio-control agent of pests in agriculture and forest ecosystems. It is also a globally important invasive insect species. To test whether dark elytra colour is associated with greater cold hardiness, we compared the survival rate of prolonged cold exposure in both yellow and black colour morphs of female and male *H*. *axyridis*. We determined the trehalose and glycogen content, trehalase activity, and the dynamics of genes associated with the trehalose metabolic pathway. Yellow forms predominated before winter began, however black forms increased from 11.15 to 30.46% after overwintering. There was no significant difference in trehalose content between the females and males during overwintering. Glycogen content in over-wintering yellow females and black males increased significantly, while it decreased in black females. Soluble trehalase activity increased significantly in all the insects except black females. Membrane-bound trehalase activity increased in black males, and decreased in black females. Trehalose and glycogen content and trehalase activity were regulated by differential expression of *TRE* and *TPS* genes. Female beetles weighed more than males and survived in low temperatures for longer periods of time, regardless of elytra colour, suggesting that mass is a stronger predictor of overwintering survival rather than colour morph. Our results provide a guide for comparing cold resistance in insects and a theoretical basis for cold storage of *H*. *axyridis* for use as natural enemies of pests in biological control programs.

## Introduction

The harlequin ladybird, *Harmonia axyridis* (Coleoptera: Coccinellidae) is an important predator of many pests, and is also one of the most effective ways to control aphids [1–3]. Therefore, it is widely used as an important biological control insect of pests in the global agricultural field. However, it is also a worldwide invasive species that causes harmful ecological impacts around the world [1, 4–5]. It is very obvious that the biggest feature of *H*. *axyridis* is the diversity of elytral colour pattern. The adult elytra are usually black (melanic) or light yellow (non-melanic) with black or red spots [6]. Colour polymorphism is controlled by a series of complex alleles that show a mosaic dominance inheritance pattern [7–8]. According to the survey, over 200 colour forms of *H*. *axyridis* are found in China, with yellow-based forms predominating. Liu reported 126 distinct morphological variants of spotted ladybirds from Maor Mountain in Heilongjiang Province [9]. In north China, the proportion of melanic and non-melanic *H*. *axyridis* adults show seasonal variation, dominated by non-melanic forms in autumn and melanic forms in spring [10]. In summary, yellow adults significantly outnumbered black adults during winter, possibly due to the relationship between climate and the formation of insect melanin [11–12]. Given that overwintering success is important for insect population growth in temperate regions [13], an important question remains whether the low temperature resistance of yellow adults is higher than that of black adults.

Trehalose is a non-reducing disaccharide that is mainly synthesized in the fat body and has a clear role in energy metabolism in insects [14–16]. In insects and yeast, trehalose is mainly synthesized by the trehalose-6-phosphate synthase (TPS or ostA) / trehalose-6-phosphate phosphatase (TPP or ostB) pathway, which is also the most widely distributed pathway in other organisms [15, 17–18]. In addition, other carbohydrates, for example, glycogen, can be converted to trehalose via the trehalose and glycogen metabolism pathways [19]. Trehalose, glucose and glycogen in insects can be interconverted through this pathway [20]. When insects experience low temperatures, starvation or other stresses, each trehalose molecule can be hydrolyzed into two glucose molecules by trehalase (TRE). Two forms of trehalase, soluble trehalase (TRE1) and membrane-bound trehalase (TRE2) with a trans-membrane structure on the N-hydroxy group have been identified and cloned in many insect species [19, 21–24].

Both trehalose and glycogen play an important role in cold hardiness or starvation resistance of insects [25–28]. In addition, trehalose is very important for the growth and development of insects [14]. It is an important energy source in the haemolymph of insects, and trehalose can be found in all developmental stages of insect [14, 29–30]. Trehalose can accumulate in response to adverse environmental conditions, including desiccation, cold, oxidation and hypoxia [25–26, 31], protecting DNA molecules, cell membranes and proteins [32–34]. It acts as an intermediate product in the process of insects resisting adverse environments [30, 35–36], as well as plays a key physiological role in short-term cold storage, especially during diapause [27, 37]. Trehalose content of diapause insects is typically higher than in non-diapause insects [38–39].

*H*. *axyridis* is known to have strong cold resistance in winter, for example, accumulating triglycerides, glycogen and other energy substances for use in cold environments [40]. A previous study has shown a >80% survival rate when *H*. *axyridis* adults were stored for 150 days at 3°C and 6°C [41]. Therefore, in the present study, we aimed to study the relationship between cold tolerance and morphological characteristics or trehalose metabolism of pre- and over-wintering populations of *H*. *axyridis*. Adults were divided into four groups of yellow females, yellow males, black females and black males. Trehalose content, glycogen content, TRE activity, and the expression level of key genes in yellow and black forms were measured during different storage periods. Body weight and survival rate (%) were assessed to understand the potential molecular mechanism of cold resistance in *H*. *axyridis*.

## Materials and methods

### Insect cultures

We collected pre-wintering *H*. *axyridis* individuals in late September and early October of each year from 2013 to 2015. In addition, *H*. *axyridis* individuals after over-wintering were collected from 30 March to 10 April 2014. All the individuals were collected from areas of high insect abundance, such as woods and orchards on Maoer Mountain of Heilongjiang Province, China (N45°20′–45°25′, E127°30′–127°34′). The location is the experimental forest farm of Northeast Forestry University, and permission was obtained from the Northeast Forestry University before collection. All subsequent experiments were performed in the laboratory and did not require a specific location. Moreover, *H*. *axyridis* used in this study is not an endangered or protected species.

The black and yellow individuals were counted to determine the locally dominant forms and the individuals were stored in breathable (small holes in the 500 mL bottles) plastic bottles, which were placed together with an ice bag in a foam incubator, and transported to the laboratory. The individuals were temporarily stored at 10°C in a refrigerator and the samples of the over-wintering population were obtained by storing pre-wintering populations in a refrigerator at 5°C for 2 months. *H*. *axyridis* individuals collected in the autumn of 2014 were used to measure changes in body weight and survival during different storage periods. The individuals collected in the autumn of 2015 were used for experiments on the effects of low temperature storage on the trehalose metabolism pathway, including the detection of trehalose and glucose content, trehalase activity and related gene expression levels.

### Morphological assessment and sex discrimination of *H*. *axyridis*

*H*. *axyridis* individuals were separated according to the background colour of the elytra into two groups: black and yellow. We further subdivided the groups into black females (BF), black males (BM), yellow females (YF) and yellow males (YM). Sex discrimination of *H*. *axyridis* was based on bent distal margin of the fifth abdominal sternite, slightly smaller body and the white base of the labrum of the male; flat abdominal sternite, larger body size, and the black base of the labrum of the female [9].

### Low-temperature storage treatments and survival analysis

The pre-wintering *H*. *axyridis* individuals collected in the autumn of 2014 were further subdivided into black females (BF), black males (BM), yellow females (YF) and yellow males (YM). About three hundred adults from each category were selected and placed in ventilated plastic boxes wrapped with newspapers to protect them from light. The plastic boxes were stored in a refrigerator at 5°C and were not fed any food. The individuals were weighed and the survival was calculated at 0 d, 5 d, 10 d, 15 d, 20 d, 40 d and 60 d, respectively. Five individuals were randomly selected from each group for weighing.

### Analysis of trehalose and glycogen content

The trehalose and glycogen contents of the pre-wintering population and the over-wintering population groups were measured. Three samples of abdominal tissue of adults were placed in a 5 mL centrifuge tube. After adding 500 μL of 20 mM phosphate buffered saline (PBS, pH 6.0) to the tube, the tissue samples were homogenised at 0°C in a homogeniser (TGrinder OSE-Y20, TIANGEN Biotech (Beijing) Co., Ltd., China), followed by sonication for 30 s in an ultrasonic processor (VCX 130PB, Sonics & Materials, Inc., USA). Homogenates were centrifuged at 12,000 × *g* at 4°C for 10 min after adding PBS (2.5 mL). Precipitates were removed and aliquots (1 mL) of supernatant were assayed to determine the amount of protein content using a protein dye-binding method (Bio-Rad Protein Assay, USA) with bovine serum albumin as standard. Then, 1 mL of supernatant was added to a 1.5 mL tube and boiled, after which the solution was centrifuged at 12,000 × *g* for 10 min to remove any residual protein. The supernatant was divided into two tubes: one was directly subjected to a glycogen content assay, and the other was processed for trehalose measurement [42].

To test for trehalose content, aliquots of supernatant (100 μL) was put into a 1.5 mL centrifuge tube, 100 μL of 1% sulphuric acid was added, and the tube was incubated in water at 90°C for 10 min to hydrolyse glycogen, after which it was cooled on ice for 3 min. The supernatant was incubated again in 90°C water for 10 min after adding 100 μL 30% potassium hydroxide solution to decompose glucose. Then 4 volumes of 0.2% (M/V) anthrone (Sigma, USA) in 80% sulphuric acid was added after it was cooled on ice for 3 min, and the supernatant was boiled for 10 min. After cooling, 200 μL of the reaction solution was placed into a 96-well plate and absorbance at 620 nm was determined using a SpectraMax M5 microplate reader (Molecular Devices, USA). Trehalose content was calculated based on a standard curve (curves drawn from standard samples of different concentrations of trehalose) and the results were expressed as nmol trehalose /μg protein. The treatments were replicated three times.

Glycogen content was measured as described by Santos [43]. The supernatant described above (200 μL) was incubated for 4 h at 37°C in the presence of 40 μL (1 U) amyloglucosidase (EC 3.2.1.3, Sigma, USA) diluted in 100 mM sodium acetate (pH 5.5) to hydrolyse glycogen. The amount of glucose generated from glycogen was determined using a Glucose Assay Kit (GAGO20-1KT, Sigma, USA) following the manufacturer’s instructions. Controls were prepared in the absence of the enzyme, and the amount of glycogen was calculated as follows: (total glucose − endogenous glucose) ∕ total protein. Finally, the result was expressed as nmol glucose /μg protein. The treatments were replicated three times.

### Trehalase enzyme activity assay

The changes in trehalase enzyme activity, TRE1 and TRE2, of the pre-wintering population and the over-wintering population groups were determined by the method of Tatun et al. [44]. Three samples of abdominal tissue of adults were homogenised in a 5 ml tube in a TGrinder OSE-Y20 homogeniser at 0°C after adding 20 mM pH 6.0 PBS (200 μL), followed by sonication for 30 s in a VCX 130PB ultrasonic processor. The homogenates were centrifuged at 1000 × *g* at 4°C for 10 min after adding PBS (2.5 mL), and cuticle debris was removed and centrifuged at 105,000 × *g* and 4°C for 60 min. The supernatant fraction was collected into a new tube and used directly to measure the activity of TRE1. The precipitate fraction was washed twice with PBS, then suspended in PBS (200 μL) for measurement of TRE2. The amount of protein in each sample was determined prior to trehalase assay using a protein dye-binding method (Bio-Rad Protein Assay) with bovine serum albumin as a standard. For the trehalase activity assay, the reaction mixture (250 μL) consisted of 62.5 μL of 40 mM trehalose (Sigma, USA) in 20 mM PBS (pH 6.0), 50 μL TRE1 or TRE2 fraction, and 137.5 μL PBS. The mixture was incubated at 37°C for 30 min, and the reaction was stopped by heating in boiling water for 5 min. Coagulated protein was removed by centrifugation at 12,000 × *g* at 4°C for 10 min, and an aliquot of the resulting supernatant was used to measure the amount of glucose using a Glucose Assay Kit (GAGO20-1KT) following the manufacturer’s instructions. Data were expressed as mg glucose/g protein/min. The treatments were replicated three times.

### Quantitative real-time PCR

We determined the relative expression levels of related genes of the pre-wintering population and the over-wintering population groups. Total RNA was isolated from two samples of abdominal tissue after low temperature storage and 1 μg total RNA was used for synthesis of first-strand cDNA using a PrimeScript RT® with gDNA Eraser kit (TaKaRa, Dalian, China). The relative expressions of seven *TRE* genes and one *TPS* gene were estimated by real-time PCR using a CFX96™ real-time PCR detection system (Bio-Rad) and SsoFast^TM^ EvaGreen® Supermix (Bio-Rad), in a 20 μL of total reaction volume containing 1 μL cDNA sample, 1 μL (10 μmol/μL) of each primer, 7 μL RNase-free and DNase-free water, and 10 μL SsoFast^TM^ EvaGreen® Supermix. To obtain reliable and valid gene expression profiles, quality assurance and controls are essential. Templates were replaced with sterile water as negative controls and the housekeeping gene *18S* rRNA was used as a reference. The primers used were: Ha18S-qF (5′-CGCTACTACCGATTGAA-3′) and Ha18S-qR (5′- GGAAACCTTGTTACGACTT-3′). Design of primers for the *TPS* and *TRE* genes of *H*. *axyridis* using Primer Premier 5 and DNA Star softwares are shown in Table 1 and the primers were verified before experimentation [45]. The gene sequences used were from the GenBank database under the accession code (HM056038, FJ501961, JX514372, KP318742, KX349223, KX349224, KX349224, and FJ501960). The cycling parameters were 94°C for 5 min for initial denaturation, followed by 40 cycles at 94°C for 15 s, and 59°C for 30 s. Melting curve analysis was performed at 65–95°C to ensure that only a single product was amplified.

**Table 1:** Primers for *TPS* and *TRE* genes of *H*. *axyridis* used in qRT-PCR.

Gene name	Primer name	Primer sequence
*HaTRE1-1*	HaTRE1-1QF	5’-CTTCGCCAGTCAAATCGTCA-3’
	HaTRE1-1QR	5’-CCGTTTGGGACATTCCAGAT-3’
*HaTRE1-2*	HaTRE1-2QF	5’-TGACAACTTCCAACCTGGTAATG-3’
	HaTRE1-2QR	5’-TTCCTTCGAGACATCTGGCTTA-3’
*HaTRE1-3*	HaTRE1-3QF	5’-ACAGTCCCTCAGAATCTATCGTC-3’
	HaTRE1-3QR	5’-GGAGCCAAGTCTCAAGCTCATC-3’
*HaTRE1-4*	HaTRE1-4QF	5’-TTACTGCCAGTTTGATGACCAT-3’
	HaTRE1-4QR	5’-CATTTCGCTAATCAGAAGACCCT-3’
*HaTRE1-5*	HaTRE1-5QF	5’-TGATGATGAGGTACGACGAGA-3’
	HaTRE1-5QR	5’-GTAGCAAGGACCTAACAAACTG-3’
*HaTRE2-1*	HaTRE2-1QF	5’-TTCCAGGTGGGAGATTCAGG-3’
	HaTRE2-1QR	5’-GGGATCAATGTAGGAGGCTGTG-3’
*HaTRE2-2*	HaTRE2-2QF	5’-CAATCAGGGTGCTGTAATGTCG-3’
	HaTRE2-2QR	5’-CGTAGTTGGCTCATTCGTTTCC-3’
*HaTPS*	HaTPSQF	5’-GACCCTGACGAAGCCATACC-3’
	HaTPSQR	5’-AAAGTTCCATTACACGCAC-3’

F: forward, R: reverse.

### Statistical analysis

**Fig 1:**
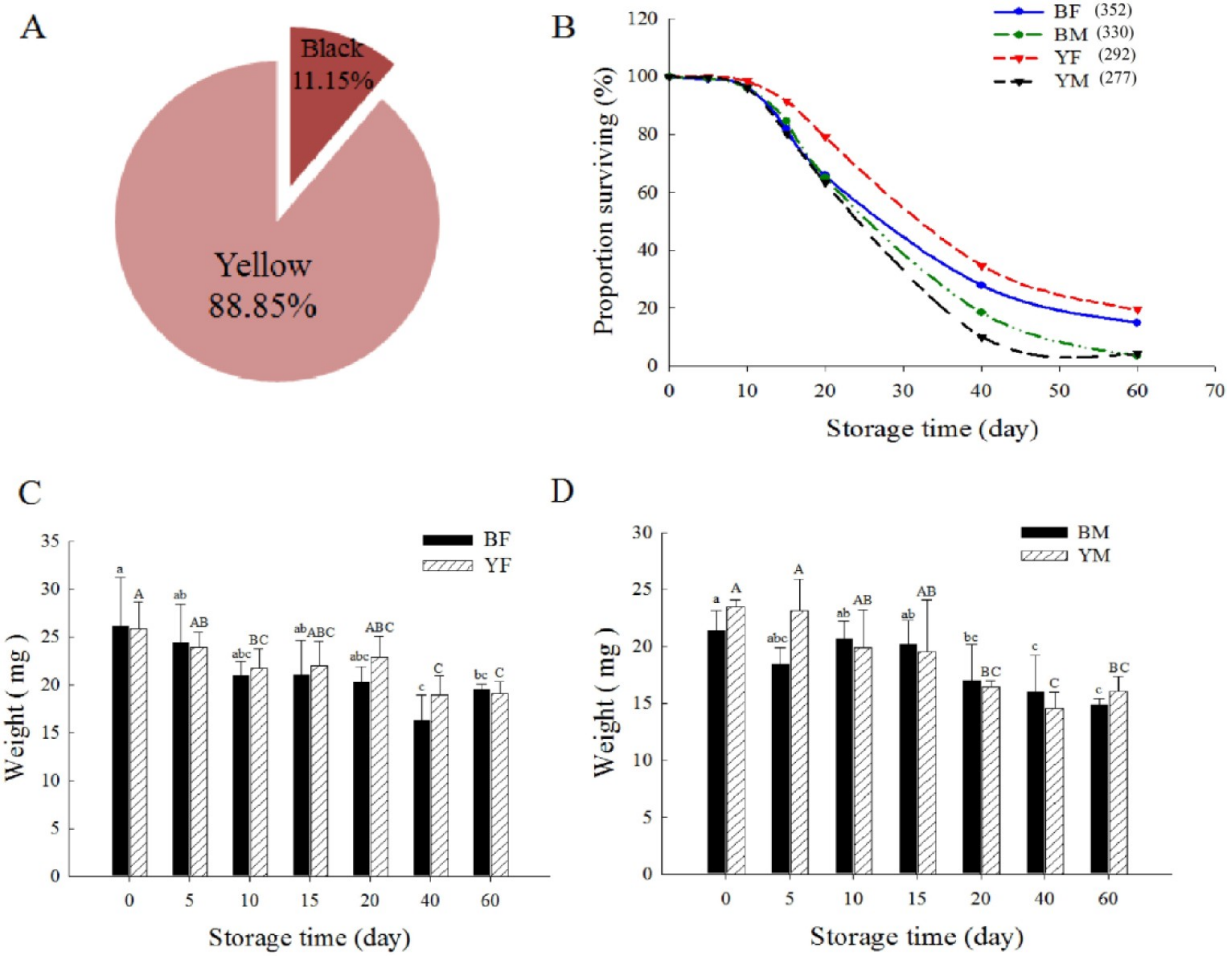
Elytral colour, surviving (%), and weight of pre-wintering *H*. *axyridis*. (A) The proportion of yellow and black individuals in late September and early October of each year from 2013 to 2015. (B) Survival of yellow (YF, Sample size: 292) and black females (BF, Sample size: 352) and yellow (YM, Sample size: 277) and black males (BM, Sample size: 330). (C) Weight of females, and (D) weight of males at different periods under low temperature storage. Each bar depicts the mean (+ SD) of three sampling dates, with different letters above the error bars denoting significant differences between means (Tukey’s test, α = 0.05; each group was analysed separately).

The CT values of several genes were determined by quantitative real-time PCR, and the average of three replicates of each sample was used for calculation (mean + standard deviation SD). Gene expression data were analysed using a relative quantitative method (2^-ΔΔCT^) [46]. Data were analysed using IBM SPSS statistics 20 software to determine the significance at P < 0.05. Tukey’s test of One-Way ANOVA was performed to test the significance of differences among treatments (**P < 0.01; *P < 0.05). Different lowercase and uppercase letters in Fig 1 indicated a significant difference at the 0.05 level following the Tukey’s test. All figures and tables were made using Microsoft Office 2013 and SigmaPlot 10.0 softwares.

## Results

### Effect of low temperature on survival and body weight of *H*. *axyridis*

We collected a total of 13,393 pre-wintering *H*. *axyridis* adults and 88.85% of pre-wintering individuals were the yellow (non-black) elytra form, and the remaining 11.15% were the black elytra form (Fig 1A). In addition, the ratio of female to male was about 2:1. From the first day to day 60, the number of surviving individuals in all groups (BF, BM, YF and YM) continued to decrease. After 60 days, it was greater for yellow females than for black females, whereas it was almost the same for black and yellow males (Fig 1B). Overall, the 20^th^ day of refrigerated was a turning point. When chilled for less than or equal to 20 days, the survival of *H*. *axyridis* was high, all were > 60% survival rate. After more than 20 days of chill, the survival was low, especially in black males and females. At the same time, weight of black females maintained a downward trend (Fig 1C). There was significant weight loss in all groups after 40 days of storage compared to before storage (Fig 1C and 1D).

### Phenotypic distribution of *H*. *axyridis* collected in spring

**Fig 2:**
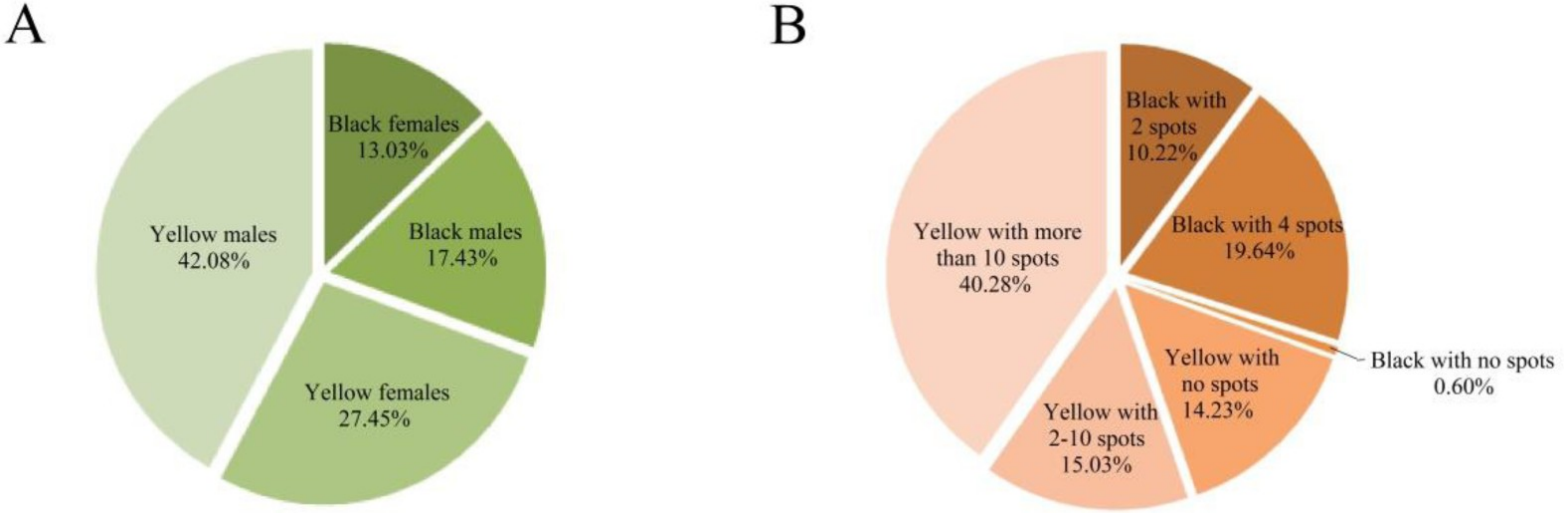
Relative distribution of elytral colours and patterns in the *H*. *axyridis* population (in late March and early April of 2014). (A) Distribution of elytral colour by sex. (B) Distribution by elytral colour and spot number.

Among the 499 individuals collected during spring in 2014, the yellow elytral colour predominated, with the highest proportion being yellow males. Black individuals (30.46% of all individuals) included those with no spots, 2 spots, and 4 spots (Fig 2B). Yellow *H*. *axyridis* included an even greater variety of spot types, ranging from 0 to 19 spots, among which individuals with >10 spots formed were the largest group (Fig 2B). There were similar proportions of yellow individuals with 2–10 spots and with zero spots.

### Trehalose and glycogen content in pre-wintering and over-wintering individuals

**Fig 3:**
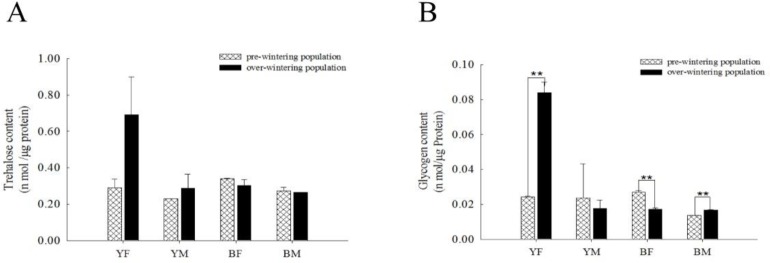
Comparison of changes in trehalose and glycogen content between pre-wintering and over-wintering *H*. *axyridis* individuals in females and males of different colour forms. (A) Changes in trehalose content, and (B) changes in glycogen content in pre-wintering and over-wintering yellow females (YF), yellow males (YM), black females (BF) and black males (BM). Each bar depicts the mean (+ SD) of three samples. Trehalose and glycogen content at the pre-wintering stage was used as a control. Asterisks show significant differences between pre-wintering and over-wintering individuals in the same group (Tukey’s test, **P < 0.01; *P < 0.05).

Compared with the pre-wintering population, there was no significant difference in trehalose content of over-wintering ladybirds (Fig 3A). Glycogen content was significantly higher (P = 0.005) in over-wintering yellow females than in pre-wintering yellow females (Fig 3). In yellow males, consistent with changes in trehalose, there was no significant difference (P = 0.711) in glycogen content (Fig 3B). Glycogen content in over-wintering black females was significantly lower (P = 0.007) than in pre-wintering black females (Fig 3B). Although there was no difference in trehalose content, glycogen content was significantly higher (P = 0.005) in over-wintering than in pre-wintering black males (Fig 3B). Overall, among the pre-wintering groups, trehalose and glycogen contents were high in black females (Fig 3). In contrast, trehalose and glycogen contents in yellow females far exceeded those of the other groups during the wintering period (Fig 3).

### Trehalase enzyme activity in pre-wintering and over-wintering individuals

**Fig 4:**
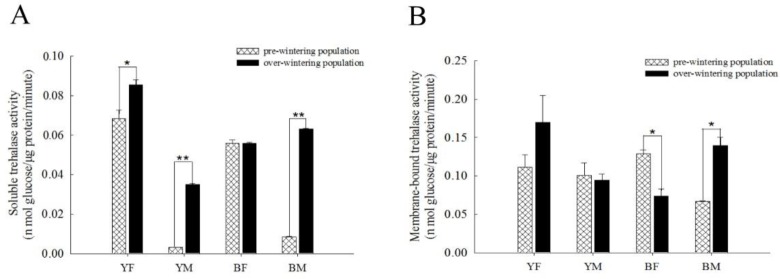
Comparison of changes in activity of two types of trehalase between pre-wintering and over-wintering *H*. *axyridis* individuals in females and males of different colour forms. (A) Changes in soluble trehalase, and (B) changes in membrane-bound trehalase in pre-wintering and over-wintering yellow females (YF), yellow males (YM), black females (BF) and black males (BM). Each bar depicts the mean (+ SD) of three samples. Trehalase activity at the stage of pre-wintering was used as a control. Asterisks show significant differences between pre-wintering and over-wintering individuals in the same group (Tukey’s test, **P < 0.01; *P < 0.05).

There were differences in TRE1 and TRE2 activity between the various *H*. *axyridis* groups during both periods. The activity of TRE1 in over-wintering yellow females was significantly higher (P = 0.038) than in pre-wintering individuals (Fig 4A). In contrast, in yellow males, only TRE1 activity was significantly higher (P < 0.01) during over-wintering than in the pre-wintering period (Fig 4A). In black females, only TRE2 activity showed a difference; in contrast to the other groups, its activity significantly decreased (P = 0.018) during the over-wintering period (Fig 4B). On the other hand, black males showed significantly increased TRE1 and TRE2 activities during over-wintering (P < 0.01; P = 0.012) (Fig 4). Yellow females showed the strongest and yellow males the weakest TRE1 activity in both pre-wintering and over-wintering periods (Fig 4). TRE2 activity was highest in black females and weakest in black males before wintering; however, among the over-wintering population, it was strongest in yellow females and weakest in black females (Fig 4).

### Analysis of relative expression level of *TRE* and *TPS* genes

**Fig 5:**
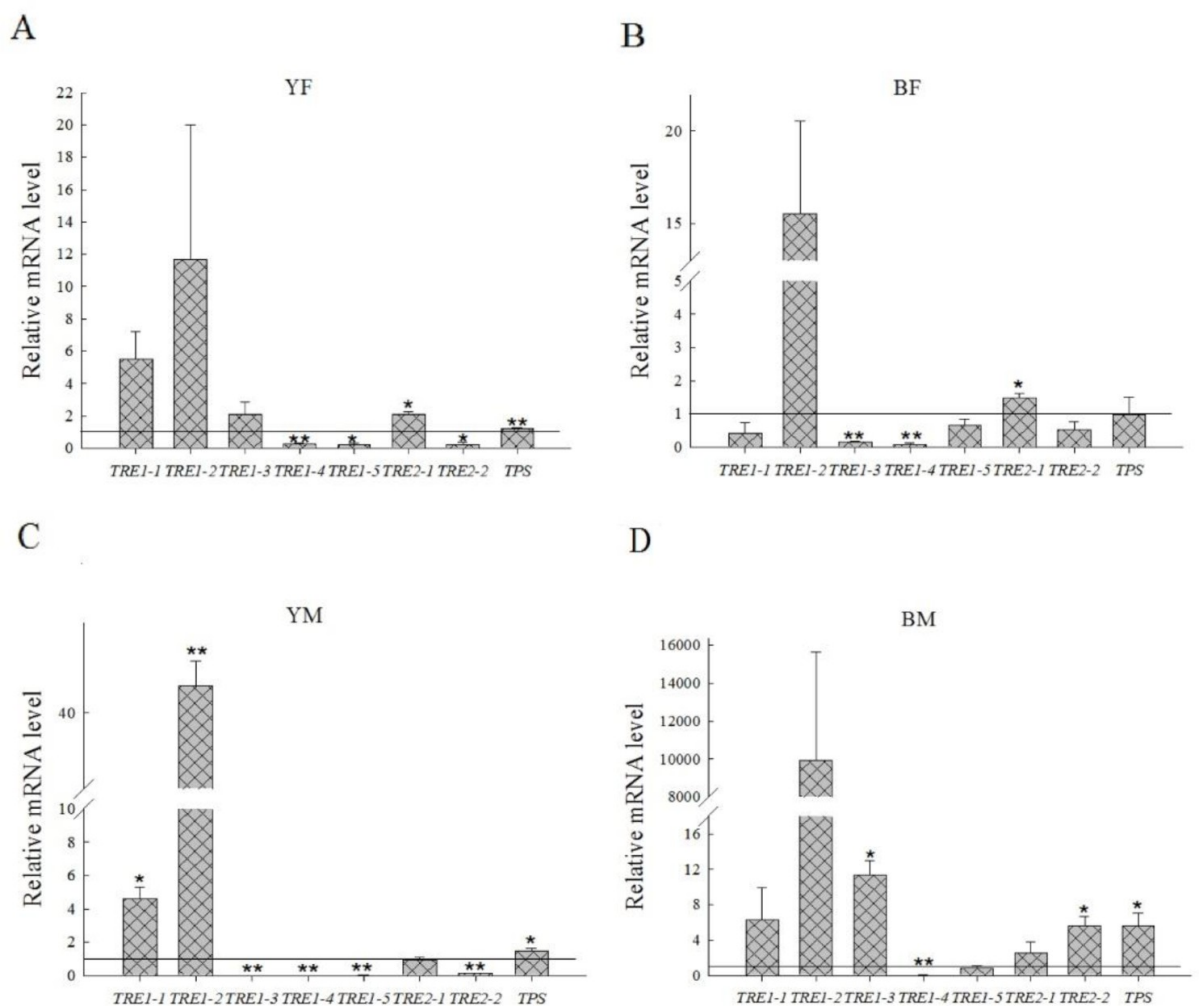
Comparison of changes in relative expression of seven TRE genes and one TPS gene between pre-wintering and over-wintering *H*. *axyridis* individuals in females and males of different colour forms. Relative gene expression is shown for yellow females (A), black females (B), yellow males (C) and black males (D). The expression level of these genes was determined with qRT-PCR using the housekeeping gene *18S* as a reference. The relative expression of each gene in the pre-wintering population was used as control. The horizontal line represents the expression level at the stage of pre-wintering. Each bar depicts the mean (+ SD) of three samples. Asterisks show significant differences in relative expression (Tukey’s test, **P < 0.01; *P < 0.05).

Analysis of relative expression levels of several genes showed that the expression of *TRE1-2* was much higher during over-wintering than the pre-wintering period for males and females of both colour forms (Fig 5). *TRE1-1* also had elevated expression (P = 0.016) in yellow males (Fig 5C). *TRE1-3* expression was significantly elevated (P = 0.011) in black males, while it was significantly reduced (P < 0.01) in black females and yellow males. The relative expression levels of *TRE1-4* and *TRE1-5* were reduced; the decrease was significant (P < 0.01) in all cases for *TRE1-4*, but only in yellow individuals for *TRE1-5* (Fig 5). *TRE2-1* expression was significantly elevated in yellow females (P = 0.010) and black females (P = 0.037), whereas *TRE2-2* expression was significantly increased only in black males (P = 0.024) and significantly reduced in all yellow individuals (P = 0.017; P < 0.01). *TPS* expression level was significantly elevated in yellow females (P = 0.003), yellow males (P = 0.045) and black males (P = 0.046) (Fig 5A, 5C and 5D).

## Discussion

### Relationship between elytral colour and ambient temperature

Cold tolerance, overwintering strategies and cold season phenotypic plasticity of *H*. *axyridis* have been studied [10, 41, 47–50]. Changes in allele frequencies of melanic and non-melanic forms in some populations suggest that melanic individuals may be advantageous in winter, but costly in summer [10, 51–53]. In addition, the ratios of elytral colour forms can vary with the season [10]. Our study shows that the proportion of non-melanic *H*. *axyridis* adults is higher than that of melanic adults in pre-wintering populations in northeast China (Fig 1). Yellow is the main elytral colour in autumn in the population we studied, similar to results of previous reports [10]. However, non-melanic forms decreased from 88.85% in autumn to 69.53% in spring, while the melanic forms increased from 11.15% to 30.46% (Fig 2). This change in relative frequencies may be related to change in ambient temperature [11–12]. Melanic forms of *H*. *axyridis* are thought to have a fitness advantage at low temperatures because their dark colour may enable them to absorb heat and increase body temperature more rapidly than non-melanic forms [5, 51, 54]. Besides, previous studies have shown that a darker cuticle can reduce rates of water loss [55–57], and increase immune function [58–59]. Our results confirm the findings of previous studies [54] and suggest that black and yellow forms of *H*. *axyridis* may have different roles in temperature or climatic adaptation.

### Relationship between sex and cold tolerance of *H*. *axyridis*

In north-east China, *H*. *axyridis* adults are known to aggregate in stone gaps or in caves to overwinter [52]. The aggregated individuals form a protective microclimate where temperatures are more stable than in the surrounding environment around them [50, 52]. Cold tolerance of over-wintering *H*. *axyridis* populations is higher than that of summer populations [60], for example, they have a lower supercooling point [50]. In addition, the pre-wintering populations can survive long time periods at low temperature [41]. A study of Labrie et al. showed that *H*. *axyridis* selected human houses as overwintering sites in Canada, and the survival of females inside houses was greater than that of males [49]. The results of the present study indicate that the differences in the survival are due to sex rather than elytral colour (Fig 1B), confirming the findings of Labrie [49]. In nature, black is good for absorbing heat from the ambient environment [11–12], which is weakened under the limitations of laboratory conditions. Zhao et al. found that the super cooling point of over-wintering black forms was lower than that of yellow forms [60–61]. Therefore, further experiments are needed in the future to verify our previous results. A decrease in survival of over-wintering *H*. *axyridis* was observed when stored at -5°C and 10°C [49]. In the study by Ruan [41], the survival of over-wintering adults collected in north-eastern China was more than 80% when stored at 3°C and 6°C for 150 days. However, the results of the present study showed that the survival was less than 10% after storage for 60 days at 5°C (Fig 1B); this was accompanied by decreased body weight (Fig 1C and 1D). Therefore, these differences in survival may be a result of differences in storage conditions, including feeding status before storage, humidity, and temperature. Female beetles weighed more than males and survived longer at low temperatures, regardless of elytra colour, suggesting that mass is a stronger predictor of overwintering survival than colour morph.

### Relationship between low temperature storage and carbohydrates

Cold acclimatization may induce accumulation of cryoprotectants, such as trehalose, glycerol, etc., while many insects over-winter by means of diapause [40]. Before wintering, insects accumulate substances such as fats, proteins, glycogen, etc., which are gradually utilized over winter. Hawakawa and Chino [62] proposed that there are at least two types of carbohydrate that accumulate in over-wintering insects: trehalose and glycogen accumulation. Glycogen and trehalose have an important relationship with insect cold resistance [62–63]. We investigated whether trehalose and glycogen content in over-wintering *H*. *axyridis* individuals were higher than in pre-wintering individuals; however, this was not consistent. There was no significant difference in trehalose content between pre-wintering and over-wintering ladybirds (Fig 3A). Glycogen content increased in yellow females and black males, while it decreased significantly in black females (Fig 3B). In an investigation on *Agonoscena pistaciae* collected from the field, total sugar, trehalose, and sorbitol increased from October to January followed by a decrease from January to March, while glycogen had the opposite trend [64], showing that trehalose and total sugars accumulated as the temperature decreased. Similarly, in *Ectomyelois ceratoniae*, trehalose, and total sugar accumulated from October to February, while glycogen content decreased [65]. Trehalose levels typically increase as the level of glycogen decreases. This may be because the glycogen is hydrolyzed and the subsequent products are incorporated into trehalose. In this experiment, we can only see an increase in trehalose and a decrease in glycogen in the YM group. It is speculated that the glycogen abundance is too low to affect the trehalose level: for example, the glycogen of the pre-wintering YF group is 0.02 nmol glucose/μg protein, which is equivalent to 0.01 nmol trehalose/μg protein. In *Pityogenes chalcographus*, trehalose and glycogen levels were highest in November and March [66]. In *Coccinella septempunctata*, diapausing individuals have higher levels of lipids, glycogen and total sugars than non-diapausing individuals, while trehalose content remained stable [67]. The content of trehalose can be increased during temperature decrease in the *H*. *axyridis* adults though it remains unclear whether it is a form of diapause in insect [26]. Du et al. found that trehalose and total sugar content of *H*. *axyridis* in the field decreased as temperature increased, while total sugar and fat content decreased step by step as storage time increased when over-wintering individuals were stored under cold conditions [68]. These results indicate that insects can accumulate trehalose and increase total sugar content under cold environmental conditions, although there may be exceptions to this based on sex or different elytral colouration. It seems that the important relationship between trehalose level and cold tolerance is not observed, but trehalose and glycogen content in black and yellow females were found to be higher than in other groups before and after wintering, respectively (Fig 3), because we only measured the trehalose and glycogen content of one stage in the middle of wintering, which is relatively incomplete and further studies are needed to explain this phenomenon.

### Relationship between low temperature storage and trehalose metabolism

Previous studies have shown that trehalose content decreased and wing development and chitin metabolism were negatively affected when TRE1 and TRE2 activities were reduced by knockdown of *TRE* genes or by trehalase inhibitor [69–71]. In the present study, TRE1 and TRE2 activities regulated the changes in trehalose in pre-wintering and over-wintering *H*. *axyridis* populations. However, there were some differences in the activities of the two TREs between yellow and black individuals, or between females and males (Fig 4). These results showed that high TRE activity did not reduce the abundance of trehalose. Therefore, it is speculated that the role of TRE in regulating trehalose abundance is not so obvious, and there may be other mechanisms involved, which requires further study. Trehalose content and TRE activity decreased with increasing storage time, but trehalose content and *TPS* expression increased significantly, while TRE1 activity decreased when *H*. *axyridis* was put into cold-stress conditions by cooling from 25°C to -5°C [26–27]. The results of the present study show that differences in TRE1 and TRE2 activities and trehalose content are accompanied by differences in the expression of *TRE* and *TPS* genes (Fig 5). Although the activity of TRE1 was enhanced in yellow males and females, the expression level of *TPS* also significantly enhanced, and their combined action resulted in no significant change in trehalose content (Figs 4A and 5). Similarly, in black males, both TRE1 and TRE2 activities were significantly enhanced, but the expression level of *TPS* was also increased (Figs 4 and 5D). Although the expression of *TPS* was not significantly affected in black females during overwintering, the expression of all seven *TRE* genes was significantly affected (some were up-regulated and some down-regulated; Fig 5B). This suggests that *TRE* genes play a key role in black females. Trehalase genes have different roles in different insect species. The seven trehalase genes in *H*. *axyridis* have a combined effect on trehalase activity in development and physiological activity [14, 26, 72]. Our results show that both *TRE1-1* and *TRE1-2* expressions contributed to TRE1 activity, while *TRE2-1* contributed to TRE2 activity in yellow individuals (Figs 4 and 5). In black males, the expressions of *TRE1-1*, *TRE1-2* and *TRE1-3* were higher and increased TRE1 activity, while expressions of both *TRE2* genes increased contributing to TRE2 activity (Figs 4 and 5D). These results are consistent with other research findings that *TRE1* and *TRE2* have different functions [14, 23, 71], and that these functions may be complementary, with the expression of one *TRE* gene increasing, while the expression of the other was inhibited [73].

Many insects undergo a cold-acclimatization process before winter, thereby increasing their cold tolerance and ability to maintain themselves under low temperature stress [74]. Moderate cold adaptation of insects prior to low temperature stress can increase survival, lower the lethal temperature, prolong the semi-lethal time LT_50_ (the time that causes 50% of individual deaths at a specific temperature), and reduce super cooling points [75–77]. Combined with our experimental results, female yellow ladybirds may be more conducive to prolonging low temperature storage time. In order to mass-produce the natural insect enemy *H*. *axyridis*, the optimal temperature for cryopreservation and the supplementary feeding of antifreeze substances should be studied in the future.

## Conclusions

This study mainly found that *H*. *axyridis* individuals with yellow (non-black) elytra predominate the autumn population, and the females have higher survival at 5°C than males, suggesting that they have stronger cold tolerance. However, the trend of trehalose and glycogen content is inconsistent between the YF and BF groups. Therefore, the link between the abundance of trehalose or glycogen and the cold tolerance of ladybird adults may not be significant. Since the present study measured the change of trehalose and glycogen content once, it is not conclusive. Further research is needed to verify whether trehalose and glycogen play a critical role in the wintering process.
